# Influence of Rice Husk and Wood Biomass Properties on the Manufacture of Filaments for Fused Deposition Modeling

**DOI:** 10.3389/fchem.2019.00735

**Published:** 2019-10-31

**Authors:** Marie-Joo Le Guen, Stefan Hill, Dawn Smith, Beatrix Theobald, Evamaria Gaugler, Abdellatif Barakat, Claire Mayer-Laigle

**Affiliations:** ^1^Scion, Rotorua, New Zealand; ^2^IATE, Univ Montpellier, CIRAD, INRA, Montpellier SupAgro, Montpellier, France

**Keywords:** 3D printing, rice husk, wood, biomass, composites, extrusion

## Abstract

Additive manufacturing or 3D printing has the potential to displace some of the current manufacturing techniques and is particularly attractive if local renewable waste resources can be used. In this study, rice husk, and wood powders were compounded in polylactic acid (PLA) by twin screw extrusion to produce filaments for fused-deposition modeling 3D printing. The biomasses were characterized in terms of physical features (e.g., particle size, density) and chemical compositions (e.g., solid state nuclear magnetic resonance, ash content). The two biomasses were found to have a different impact on the rheological behavior of the compounds and the extrusion process overall stability. When comparing the complex viscosity of neat PLA to the biomass/PLA compounds, the integration of wood powder increased the complex viscosity of the compound, whereas the integration of rice husk powder decreased it. This significant difference in rheological behavior was attributed to the higher specific surface area (and chemical reactivity) of the rice husk particles and the presence of silica in rice husks compared to the wood powder. Color variations were also observed. Despite the biomass filler and rheological behavior differences, the mechanical properties of the 3D printed samples were similar and predominantly affected by the printing direction.

## Introduction

The popularity of incorporating lignocellulosic biomasses in thermoplastic polymers has increased significantly due to their sustainability advantage, low density, thermal, and noise insulation capacity and stiffening performances (Le Guen and Newman, 2007; Müssig, 2010; Gallos et al., 2017; Lammi et al., 2018). The properties of the composites are related to (a) the amount of biomass incorporated and (b) the interactions between the polymeric matrix and the lignocellulosic particles in terms of physical (e.g., surface area, aspect ratio) and chemical interactions (e.g., interface compatibilisation). Amongst the lignocellulosic feedstocks available, rice husk is of particular interest due to its current abundancy (i.e., estimated between 128 and 148 million tons of unutilized waste) (Giddel and Jivan, 2007; Pode, 2016), and chemical make-up. It is composed of around 80–85 wt.% lignocellulosic materials (e.g., cellulose, hemicellulose, lignin), 15–20 wt.% of amorphous silica and 1.1 to 2.5 wt.% of proteins (Juliano et al., 1987; Vadiveloo et al., 2009; Ummah et al., 2015). Previous research studies have reported its potential applications in composite structures (Kumar et al., 2012), as an energy feedstock (Chungsangunsit, 2009) or amorphous silica feedstock (Yalçin and Sevinç, 2001). In parallel, the incorporation of nano-silica in polylactic acid (PLA) was also reported to improve the thermal stability and mechanical properties of composites (Lv et al., 2016).

3D printing (3DP) or additive manufacturing is an emerging technology that enables the creation of innovative designs and the combination of materials that were previously impossible or impractical to make. The incorporation of fillers in 3DP has demonstrated that the filler's physical features could be transferred to the 3D printed object (Montalvo Navarrete et al., 2018; Zeidler et al., 2018). These attractive traits have been demonstrated with wood (Mirko et al., 2016; Tao et al., 2017), clays (Revelo and Colorado, 2018), metals (Gibson et al., 2018), and seashells (Graichen et al., 2017; Singamneni et al., 2018a; Zeidler et al., 2018).

The combination of (a) the inherent properties of polymer-filled compounds and (b) the control of the 3D printing process directionality, leads to new functionalities to printed objects (Liles et al., 2018; Wang et al., 2018). Examples of such functionalities for lignocellulosic feedstocks include hydromorphic properties (Le Duigou et al., 2016; Sydney Gladman et al., 2016), electrical conductivity (Shao et al., 2018) and vibration damping (Zeidler et al., 2018).

In this present research, the influence of rice husk powder integrated into a PLA matrix is investigated for fused deposition modeling (FDM) 3DP applications, and compared to wood flour PLA 3D prints. The impact of the two biomasses during the extrusion compounding process, and on the mechanical properties of 3D printed rectangular parallelepipeds are observed and quantified.

## Materials and Methods

### Biomasses and Polymer

Carmargue rice (*Oryza*) husks were supplied from Silo des Tourelles- C*omptoir Agricole du Languedoc* (Aigues Mortes, France). The husks were first sieved with a screen of 0.8 mm using a vibratory sieve shaker 400 (Ritec, France) to remove soil dust and stones. The husks were subsequently milled in a vibratory ball mill model DM-1 (Sweco®, USA) consisting of a 36 L abrasion-resistant elastomer grinding chamber filled with 25 kg of ceramics cylinders (diameter and length of 13.5 mm) and 25 kg of ceramics balls (diameter of 13.5 mm). The chamber's motion was controlled by a vibrating mechanism composed of high-tensile steel springs. One kilogram of rice husk was placed in the chamber and milled for 10 h to obtain fine rice husk powder. The ground powder was dried in an oven at 60°C for 12 h and stored in aluminum foil vacuum packaging bags prior to utilization.

Wood flour (*Pinus radiata* D. Don) was supplied by a plywood mill (Carter Holt Harvey, Kinleith Mill, Tokoroa, New Zealand). Only the particles below 125 μm were used after separation using a 43305 F motorized sieve shaker (Humboldt, USA) and 125 μm mesh sieve (Endercotts, UK). The wood flour powder was dried at 100°C for at least 24 h prior to extrusion.

PLA 3001D was obtained from Nature works (USA) and dried overnight prior to extrusion. The PLA and PLA compounds were dried for at least 4 h at 55°C prior to extrusion.

### Processing

#### Compounding

The filaments were made in two sequential steps: (a) compounding the polymer and biomasses at 10 wt.% into pellets and (b) extrusion of the filament for FDM. Both steps were carried out on a 26-mm scientific twin screw, co-rotating extruder LTE26-40 (Labtech Engineering Ltd, Thailand), which had a 40 L/D ratio and was fitted with a vacuum crammer. During Step (a), materials were hand-mixed in plastic bags and fed into the volumetric feeder. The speed of the feeder was set to 20 rpm. The extruder screw speed was set to 200 rpm. The temperature was set to 190°C at the first 5 zones and 200°C at the last 5 zones. Pellets were cut to around 2 mm in length. During Step (b), the settings of the twin-screw extruder were modified (Table 1) and the pelletizer replaced by a winding unit set to produce a 1.75 mm diameter filament.

**Table 1:** Extrusion settings for Step (b) to manufacture the FDM filaments.

**Temperature (^°^C)**	**160, 170, 170, 170, 180, 180, 190, 190, 200, 200**
Screw speed (rpm)	200
Feed speed (rpm)	8
Speed of haul-off unit (rpm)	8–13

#### 3D Printing

The objects were printed on a M2 fused deposition modeling printer (Makergear, USA) fitted with a 0.75 mm die nozzle. The platform temperature was set at 70°C and the nozzle temperature at 210°C. Rectangular beams (60 × 10 × 2.5 mm) for flexural testing were printed using slicing software (Simplify 3D®, USA) based on 0.2 mm layers thickness and no strand overlap. The 3D printing direction was carried out at either 0° or 90° of the beam length.

### Characterization of the Biomasses

#### Physical Characterization

##### Particle size distribution

The particle size distribution of the risk husk powder and wood flour powder were measured by a laser diffraction particle size analyzer Hydro 2000S (Malvern Instruments Ltd., UK) with distilled water/ethylic alcohol solution (50:50 in volume) to allow good powder dispersion whilst avoiding swelling. The results are expressed using the Mie method with a refraction index of 1.53 corresponding to those of sawdust particles (Malvern, 2007). The results are generated in volume, based on the assumption that the particles are spherical. Five replicates were carried out for each biomass and the mean average distribution is reported.

##### Scanning electron microscopy (SEM)

Rice husk and wood powders were imaged with a scanning electron microscope Phenom Pro-X (Phenom, France) using a charge reduction sample holder and an acceleration voltage of 5 kV (wood powder) and 10 kV (rice husk powder), respectively.

##### Tapped density and apparent density

The apparent and tapped density was measured in duplicate using a standard tapped density analyzer (Autotap, Quantachrome instrument®, USA). The experiment consisted of filling a graduated test tube with 200 mL of powder and submitting it to 1,500 taps. The apparent and tapped densities were calculated as the ratio of the mass of the powder to the initial and final volume respectively.

#### Chemical Characterization

##### pH measurements

One gram of biomass powder was stirred into 15 mL of distilled water at room temperature. Four pH measurements over 4 h were recorded using a ph meter (Mettler Toledo, USA). The four measurements were then averaged.

##### Solid-state nuclear magnetic resonance (NMR) spectroscopy

Solid state CP-MAS (Cross Polarization-Magic Angle Spinning) ^13^C Nuclear Magnetic Resonance spectra were acquired at 50.3 MHz on a Bruker Avance III 200 spectrometer (Bruker BioSpin, Germany) fitted with a 4 mm solid state MAS probe (Bruker BioSpin, Switzerland). The samples were packed into a 4 mm rotor and spun at 5 kHz. Each 3 μs, a 90° proton preparation pulse was followed by a 1 ms contact time and a 30 ms acquisition time, with proton decoupling, and a 1.5 s recycle delay. All spectra were calibrated so that the cellulose Iβ interior C4 peak was assigned a value of 89.3 ppm relative to tetramethylsilane (Newman et al., 1993).

##### Ash content

Around 2 g of powder was first dried in an oven at 130°C during 90 min and weighed to determine the dry mass (m_s_). The sample was subsequently heated at 900°C for 2 h and re-weighted (m_r_) after cooling in a desiccator to room temperature. The ash content was calculated according to:

(1) $$\begin{array}{r} Ash=100\cdot {\text{m}}_{\text{r}}/{\text{m}}_{\text{s}} \end{array}$$

Where:

*Ash* is in percentage,

m_s_ is the sample dry mass in grams,

m_r_ is the sample residual mass in grams.

Experiments were carried out in triplicate and averaged.

##### Chemical composition

Carbohydrates and lignin content of the biomasses were measured after concentrated acid hydrolysis. The lignin content in samples was determined by the Klason method. Briefly, 100 mg of dried samples were treated with 72% v/v H_2_SO_4_ at ambient temperature for 2 h. The solutions were diluted with water to 12% v/v H_2_SO_4_ and autoclaved at 100°C for 3 h. The hydrolysates were filtered (10 μm) and the Klason lignin content was determined as the weight of the residue after drying at 105°C for 24 h. The monomeric sugars glucose, xylose, and arabinose were determined using high-pressure liquid chromatography (HPLC). A Waters system, using a BioRad HPX-87H column at 40°C and 0.3 mL/min was used for analysis. All analyses were performed in triplicate.

### Characterization of the Biomass Compounds

#### Rheology

Complex viscosity of the neat PLA and the compounds was measured on a AR 2000 rheometer (TA instruments, USA). The experiments were recorded in triplicate in frequency sweeps from 100 to 1 Hz at 200°C and 1% strain. The linear visco-elastic region was determined at 1 Hz.

#### Molecular Weight

Gel Permeation Chromatography (GPC) experiments were carried out on a using a styrene divinylbenzene (SDVB) copolymer gel column set consisting of 2 analytical columns (SDV Lux Lin M; 5 μm; 300 × 8 mm; PSS Polymer Standards Service GmbH, Germany) and a precolumn (SDV analytical precolumn, 5 μm; 50 × 8 mm; PSS Polymer Standards Service GmbH, Germany). The flow rate was set to 1 mL/min, the system was conditioned to 30°C and an injection volume of 100 μL was used. Samples were prepared in triplicate by dissolving 6 to 8 mg of sample in chloroform to a concentration of 2.0 mg/mL overnight. The solutions were filtered through a 0.45 μm PTFE filter. The instrument (Knauer, Germany) was calibrated using 12 polystyrene standards (ReadyCal Polystyrene standards; PSS Polymer Standards Service GmbH, Germany) ranging from 370 to 2,520,000 g/mol. A conventional calibration curve was established by measuring the elution volume of the standards and plotting it against the logarithm of the molar mass. The calibration data was fitted with a fourth order polynomial function (*r*^2^ = 1.00). Number average molecular weight (Mn), weight average molecular weight (Mw) and polydispersity index (PDI) were calculated from the detector response using the WinGPC UniChrom software (PSS Polymer Standards Service GmbH, Germany).

#### Dynamic and Quasi-Static Mechanical Test

Specimens for dynamic mechanical thermal analysis (DMTA) and mechanical analysis in flexural mode were tested in 3-point bending mode on a RSA-G2 DMTA (TA instruments, USA) using a 40-mm span. Specimens were conditioned for 48 h at 23°C (±2°C) and 50% (± 5%) relative humidity.

Dynamic testing was carried out from 23 to 80°C at a rate of 5°C/min. Strain oscillations of 0.1% at a frequency of at 1 Hz were applied. The storage modulus (E') and tan δ were recorded throughout the test. Each formulation and printing direction was tested in triplicates.

Flexural testing was carried out on an Instron 5566 universal testing machine (Instron, USA) fitted with a 10 kN load cell at a rate of 2 mm/min. Flexural modulus, strength and strain were recorded throughout the test. At least 5 samples for each formulation were tested.

## Results

### Characterization of the Biomass Particles

**Figure 1:**
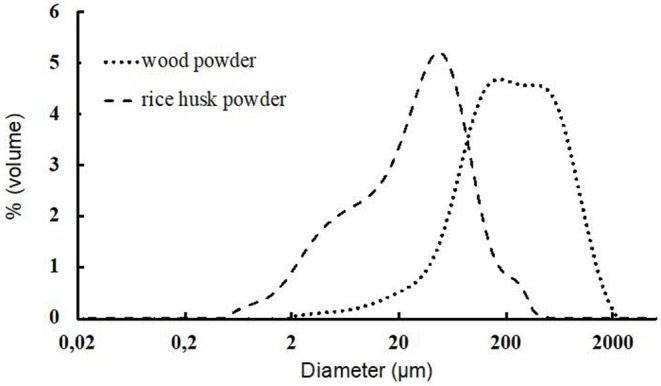
Particle size distribution for the wood powder (dotted line) and rice husk powder (dashed line).

The wood powder particles were larger than those of the rice husks with a median size of 209.9 μm for the wood powder and 28.1 μm for rice husk powder (Table 2). Both powders exhibit a widespread distribution with a similar SPAN around 3.3. SPAN expresses the width of the distribution (D90-D10/D50). Rice husk powder contains a considerable proportion of fine particles below 20 μm whereas wood powder has a high content of coarse particle (above 400 μm) as observed in the shape of the particle size distribution graph (Figure 1). As a result, the specific surface area calculated from the particle size distribution is approximately 9 times higher for rice husk powder than for wood powder. However, the particle size distributions must be interpreted with caution as laser diffraction model calculations assume spherical homogeneous particles (Bohren and Huffman, 1983).

**Table 2:** Physical characterization of the wood and rice husk powders including particle size distribution indicators, specific surface area and densities (D10: 10th percentile, D50: Median Size, D90: 90th percentile).

	**Wood powder**	**Rice husk powder**
D10 (μm)	48.4	3.5
D50 (μm)	209.9	28.1
D90 (μm)	748.1	95.2
Specific surface area SPAN ((d90-d10)/d50)	0.074 3.26	0.65 3.33
Apparent density (×10^−2^ kg.L^−1^)	8.2	45.2
Tapped density (×10^−2^ kg.L^−1^)	10.0	56.9

**Figure 2:**
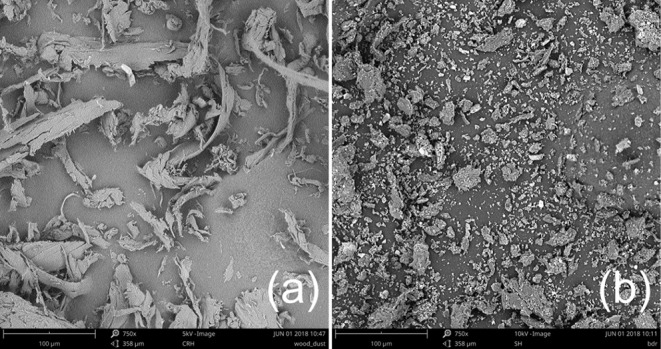
SEM images of wood powder **(a)** and rice husk powder **(b)**.

When looking at the biomass micro-morphology by SEM, large fibrous structures, >125 μm in length, are visible in the wood flour in contrast with low aspect ratio particles of the rice husk particles which supports the laser diffraction measurements (Figure 2). The presence of such particle sizes despite the sieving step is explained by their high aspect ratio that allowed them to pass through the mesh. The size and morphological differences contribute to explain the lower apparent density of the wood biomass through a lesser packing efficiency.

In terms of chemical compositions determined by HPLC, the two biomasses are consistent with literature for lignocellulosic feedstocks (Table 3) (Müssig, 2010). The main differences between powders are (a) higher lignin content for the wood powder and (b) higher ash content for the rice husk powder (i.e., 15.7 wt.% compared to 0.16 wt.% for wood). The higher ash content in rice husks is explained by the presence of silica and contributes to the five-fold difference recorded in tapped density (Table 3) (Hamdan et al., 1997). The lack of a 100 % mass balance was attributed to the presence of water soluble extractives. Those were found to be present in higher quantities in wood powder than in rice husk powder.

**Table 3:** Chemical composition in mass percentage of the biomass.

	**Wood powder**	**Rice husk powder**
Hemicellulose	17.1 ± 0.8	21.8 ± 0.7
Cellulose	44.3 ± 1.0	40.6 ± 1.0
Lignin	28.0 ± 0.8	19.4 ± 1.5
Ash (minerals)	0.16 ± 0.1	15.7 ± 0.7
Total	89.6 ± 2.7	97.5 ± 3.9

**Figure 3:**
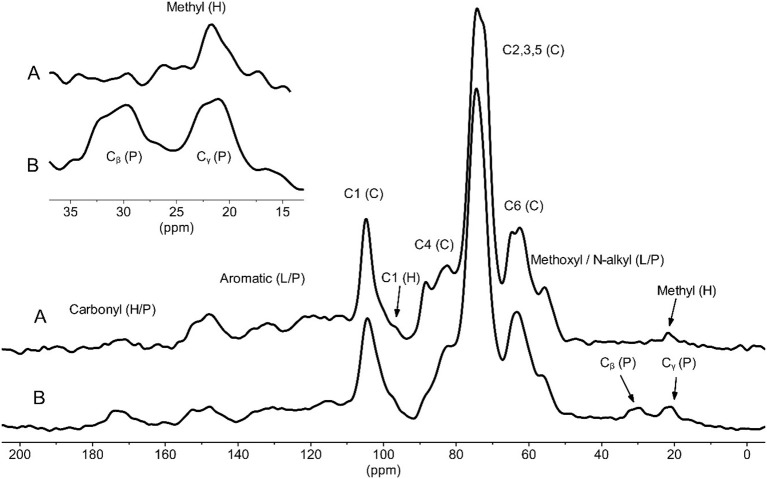
Solid-state NMR of the wood powder **(A)** and rice husk powder **(B)**. P, protein; H, hemicelluloses; L, lignin; C, cellulose. Top left spectrum is a zoom in of the 10–40 ppm range.

The chemical compositions of the powders as determined by ^13^C solid-state NMR were consistent with lignocellulosic biomass. The major peaks observed in the wood powder were attributed to cellulose (C1-C6), lignin methoxyl (56 ppm) and aromatic/phenolic groups, with minor contributions from hemicelluloses (Figure 3A) (Gil and Neto, 1999). The rice husk powder showed similar signals with the addition of peaks attributed to amide carbonyls (~175 ppm) and signals predominantly from the C_β_/C_γ_ amino acid side chains (C_γ_ 15-25 ppm and C_β_ 25-35 ppm) overlapping with signals from acetylated hemicelluloses (Figure 3B) (Bruker BioSpin, 2018). The amino acid signals were attributed to the known protein content present in rice husk (Juliano et al., 1987; Vadiveloo et al., 2009).

### Extrusion Process Stability

**Figure 4:**
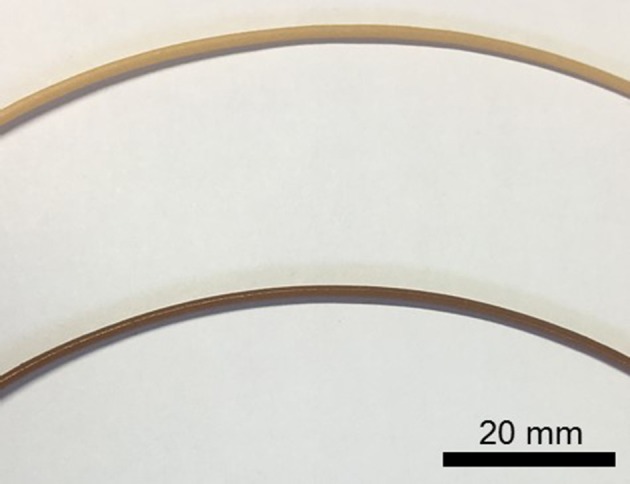
Photographs of the wood flour (top) and rice husk (bottom) FDM filaments.

During the compounding step, the die pressure was significantly different between the wood and the rice husk powders. It ranged from 68 to 73 bars and 22 to 24 bars for the wood and rice husk compounds, respectively. Low die pressure, substantial die surge and color change were observed during rice husk compounding. The processing differences were initially attributed to the particle variation in apparent densities and sizes, however, it did not account for the variation in colors. It is known that compounding lignocellulose biomass in polymer can oxidize the carbohydrates contained in the biomass and lead to a darkening of the compounds depending on the processing conditions (i.e., high shear, high temperature) (Muniyasamy, 2013; Gallos et al., 2017). Yet the variation in color between the raw feedstocks and the compounds was more intense for the PLA rice compound than those of the PLA wood, and accentuated in the FDM filament (Figure 4). Ball milling of lignocellulosic biomass during an extended period (i.e., >120 min) is known to increase surface area, decrease cellulose crystallinity and decrease the carbohydrate's degree of polymerization that consequently increases the particle surface reactivity (Vaidya et al., 2016; Gao et al., 2017). This reactivity could explain the difference of coloration caused by chemical reactions triggered by the rice husk compared to the wood powder.

During the FDM filament manufacture, the FDM rice husk filament exhibited a lower melt strength and material surged at the die causing unevenness in the filament shape. These processing effects made the rice husk filament more difficult to manufacture.

### Influence of the Biomass on the PLA

**Figure 5:**
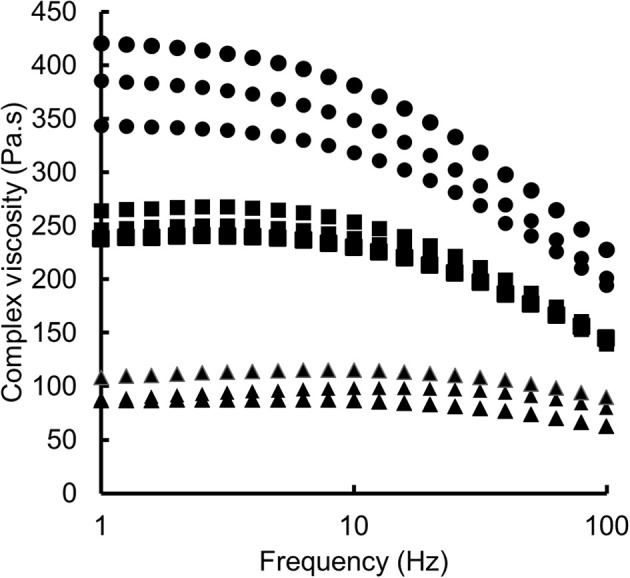
Complex viscosity as a function of frequency for: PLA (Square), PLA-wood (Circle) and PLA-rice (Triangle). Triplicate measurements were carried out and all results are presented on the graph.

In terms of rheology, the measurement of the complex viscosities of PLA and the PLA/biomass compounds as a function of frequency indicated that (a) the PLA-wood compound has a higher viscosity than neat PLA and that (b) both are exhibiting shear thinning behaviors (Figure 5). The findings are consistent with literature on biomass-filled polymers and the general effect of biomass in polymer melt (Li and Wolcott, 2004; Bettini et al., 2013; Gallos et al., 2017; Chun et al., 2018). Conversely, the rice compound was half the complex viscosity of neat PLA and indicated a poor reproducibility (Figure 5). It was hypothesized that the rice husk particles acted as a solid lubricant in the polymer matrix. However, the lack of reproducibility also suggested additional effects such as polymer hydrolysis of the backbone.

**Figure 6:**
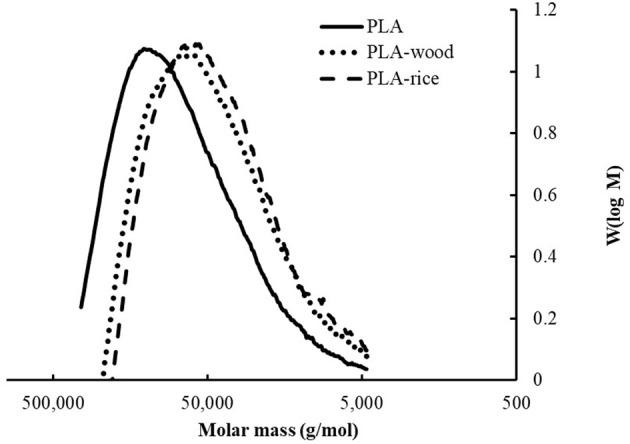
Molecular weight distribution profiles of neat PLA (solid line), PLA-wood (dotted line) and PLA-rice (dashed line) compounds.

When comparing the PLA Mn and Mw of the biomass compounds, the GPC analysis indicated no significant difference between the two fillers (Table 4). More specifically, the GPC analysis gave a Mw and Mn of 98,020 g/mol and 52,473 g/mol for the neat PLA. After extrusion with biomass powders, significantly lower average molecular weights were recorded, i.e., Mw = 66,790 g/mol and Mn = 33,780 g/mol for PLA-wood and Mw = 59,853 g/mol and Mn = 31,463 for PLA-rice (Table 4, Figure 6). This finding is consistent with literature, as PLA is known to hydrolyse in the presence of moisture or acid groups in the biomass during extrusion (Le Guen et al., 2017; Thumm et al., 2018). This translated to an average molecular weight decrease of between 32 and 39% for the PLA compounded with wood and rice husk powders. No significant difference was observed between fillers. The reduction was also observable in the molecular weight distribution (MWD) profiles (Table 4). Similarly, no significant changes in the polydispersity indices (PDI) of pure PLA compared to PLA with biomasses were observed (Table 4).

**Table 4:** Molecular weight values and polydispersity indices of the PLA neat and compounded with biomasses.

	**Mn (g/mol)**	**St. Dev. Mn (g/mol)**	**Mw (g/mol)**	**St. Dev. Mw (g/mol)**	**PDI (–)**	**St. Dev. PDI (–)**
PLA	52,473	5,245	98,020	6,294	1.9	0.24
PLA-wood	33,780	2,735	66,790	2,776	2.0	0.08
PLA-rice	31,463	3,526	59,853	5,963	1.9	0.03

*n = 3*.

When comparing the effect of the fillers onto PLA, the GPC analysis indicated that both resulted in similar level of PLA hydrolysis, hence, the difference rheological behavior could not be attributed to a difference in Mn/Mw.

### Mechanical Performances of the 3D Prints at 0° and 90°

**Figure 7:**
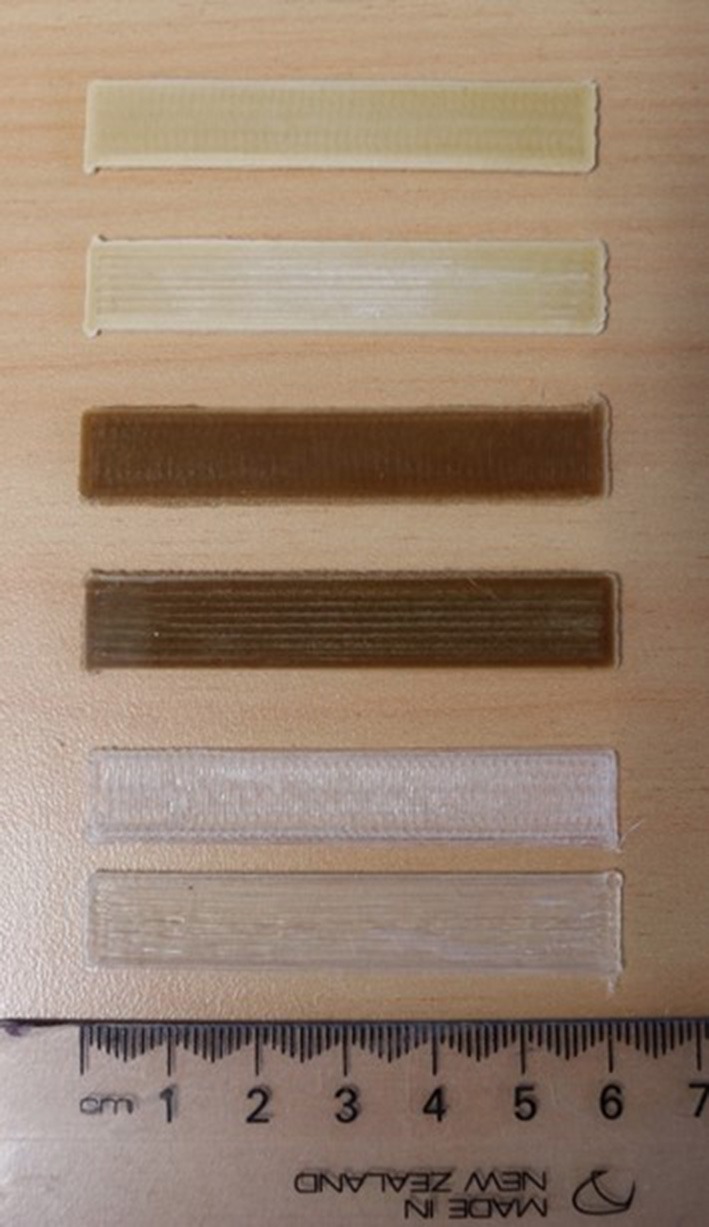
Typical 3D printed rectangular beams for flexural testing. From bottom to top PLA 0°, PLA 90°, PLA-rice 0°, PLA-rice 90°, PLA-wood 0° and PLA-wood 90°.

**Figure 8:**
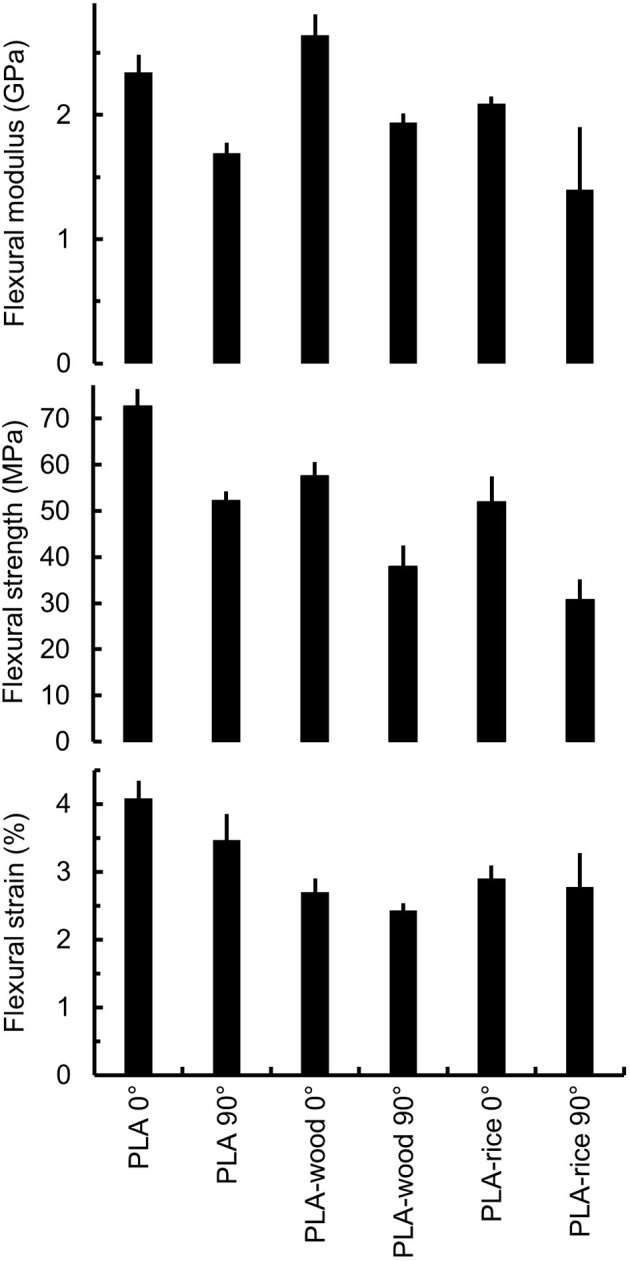
Mechanical properties comparison of the 3D printed beams PLA, (**Top**) Flexural modulus (**Middle**) Flexural strength and (**Bottom**) Flexural strain (Error bars indicate the ± 95 % confidence interval).

The infill of the print direction was carried out at 0° and 90° of the length of the rectangular beam (Figure 7). The printing direction influenced the mechanical properties of all formulations, showing a decrease of flexural modulus and strength for the 90° printed beams (Figure 8). The anisotropy induced difference in properties between the lengthwise and widthwise printing direction that ranged between 28 and 41% variation in flexural strength, 4 to 15% in strain and 27 to 33% in modulus. Similar mechanical properties results were reported for 3D printed PLA wood rectangular beam where the printing direction (i.e., 0° and 90° of the longitudinal axis of the beam) influenced the tensile stiffness and strength by 20 and 35%, respectively (Le Duigou et al., 2016). They suggested that the mechanical properties differences between the lengthwise and widthwise printing directions were due to the lack of interlayer interactions.

No significant difference was found between the wood and the rice husk fillers in terms of mechanical property except for the flexural stiffness in the lengthwise direction where the PLA-wood sample's modulus is ~25% higher than the modulus of the PLA-rice samples. The decrease in strength compared to neat PLA and the lack of difference between the PLA-wood and PLA-rice compounds, indicated that the mechanical properties are effectively driven by the PLA inter-strands coalescence as observed by others (Le Duigou et al., 2016). This coalescence is expected to decrease with the introduction of a solid filler that hinders the strand cohesion and creates weak points within the printed object. Singamenni et al., reported a gradual loss of inter-strand and inter-layer coalescence surface, measured by optical microscopy, with increasing amount of wood flour from 5 to 20 wt.% in polybutyrate-adipate-terephthalate–polymer matrix (PBAT) (Singamneni et al., 2018b). They attributed the decrease in flexural properties to the lower strands coalescence (98% coalescence for 5wt.% filler and 64% for 20wt.%). Their findings in terms of flexural strength are consistent with those presented in this study, where the addition of either filler decreases the flexural strength of the printed samples. However, their results relative to the flexural modulus differs. They reported a decrease in flexural modulus at 10 wt.% wood fraction whereas the present results indicate an increase of flexural modulus for the PLA-wood and decrease for the PLA-rice.

The difference was attributed to the polymer matrix as PBAT is more elastomeric than PLA but it raises the possibility of synergetic/competing effects between the type of filler and polymer, leading to different mechanical behavior of the 3D printed object. Similar findings were reported by Badouard et al. who compared the reinforcing ability of flax fibers in different biopolymer matrices (Badouard et al., 2019).

The dynamic thermo-mechanical behavior of the samples tested from 23 to 80°C indicated a single transition, typical of the glass transition temperature of PLA samples (Table 5).

**Table 5:** Storage modulus values at 30 and 75°C, and glass transition temperature (Tg) for PLA, PLA-wood, and PLA-rice samples printed at 0 and 90° of the longitudinal direction.

	**PLA 0^**°**^**	**PLA 90^**°**^**	**PLA-wood 0^**°**^**	**PLA-wood 90^**°**^**	**PLA-rice 0^**°**^**	**PLA-rice 90^**°**^**
E' at 30°C (GPa)	2.66 (0.22)	1.90 (0.15)	2.31 (0.07)	1.73 (0.16)	2.50 (0.15)	2.01 (0.43)
Tg (°C)	65.0 (0.35)	65.5 (0.35)	64.7 (1.7)	65.4 (1.8)	65.3 (0.9)	64.8 (0.4)
E' at 75°C (MPa)	5.6 (0.5)	3.9 (1.6)	12.8 (2.7)	7.8 (1.1)	8.6 (1.7)	13.4 (1.2)

*The number in brackets is the standard deviation for n = 3*.

The stiffness variation due to the printing direction anisotropy agreed with the quasi-mechanical tests for the neat PLA and filled samples. Storage modulus (E') values indicated a decrease of 28.7, 25.1, and 19.7 % between 0° and 90° printing direction for respectively, the unfilled PLA, PLA-wood and PLA-rice samples, respectively (Table 5). The printing direction had no thermo-mechanical effect (e.g., Tg variation, thermal stress relaxation). However, the filler's contribution to the stiffness could be observed past the glass transition temperature where the storage modulus of the PLA-wood and PLA-rice samples was higher than the unfilled PLA samples. The retention of storage modulus indicated opposite trends between the PLA-wood samples 0° and 90°, and the PLA-rice samples 0° and 90°. Indeed, past the Tg, the wood filled PLA samples displayed a 39% difference in favor of the longitudinal printing direction. Conversely, the rice-husk filled samples displayed an unexpected 56% difference in favor of the transversal printing direction. The former finding is explained by an alignment of wood particles in the printing direction caused by shear forces in the nozzle (Sydney Gladman et al., 2016). The latter finding is more difficult to explain. It was here tentatively attributed to the smaller size of the rice husk particles and their better distribution within the PLA. The rice husk particles are around 10 times smaller than the wood ones (Figure 2, Table 2). Considering the low aspect ratio of the rice husk particles, it is conceivable that no or little alignment for the rice husk particles occurred. The difference in behavior between the rice husk and the wood was tentatively attributed to the finer particles which created a more uniform and stiffening network. This characteristic was only observed in the widthwise direction as the predominant factor (i.e., the alignment of the polymer chains and fillers) was potentially not present to mask it.

## Discussion

Overall, the mechanical behavior of the 3D printed beams at room temperature was governed by the printing direction and the polymer physical properties rather than the type of fillers being used. However, when comparing the wood powder to the rice husk powder as a filler for 3D printing in PLA, particular observations were made on the interaction between the biomasses and PLA.

### Process Stability and Thinning Behavior

Whilst both biomass fillers caused a reduction in the PLA molecular chain length, the extrusion process was more unstable during rice husk powder compounding. GPC analysis indicated that the degree of hydrolysis during processing was slightly higher for the rice husk powder than the wood powder, however the difference was not significant and could not alone justify the rheological differences. The complex viscosity analysis indicated a dramatic thinning effect of the rice husk particles on the PLA (~ 2.5 times lower at 1 Hz). Thinning/plasticization additives is fairly common in polymer processing however, it is much less common in the case of solids in the 10 to 100's micron size range. Coppola et al. found that the addition of 3 and 5 vol.%, powdered hemp shives in PLA reduced the complex viscosity of the compound compared to neat polymer (Coppola et al., 2018). This behavior was attributed to chemical degradation of the pectin contained in hemp shives. Conversely, Cipriano et al. reported the same effect on PLA caused by magnesium silicate particles (Cipriano et al., 2014). They attributed this lubricant effect to a physical interaction between the particles and the polymer chains.

In this study, both chemical and physical phenomena are valid. Rice husk is composed of 15.2 wt.% amorphous silicate which contributes to its higher density compared to wood or other lignocellulosics (e.g., flax) and could contribute to the rheological thinning behavior as reported Cipriano et al. (2014). However, the concurrent presence of organic materials (e.g., carbohydrates) in the particles also points to a chemical triggered mechanism. The difference in chemical reactivity between wood and rice powders was attributed to the particles size and the ball milling. Ball milling of biomass is known to break down the carbohydrates which increases their reactivity (e.g., better enzymatic adsorption) (Vaidya et al., 2016; Gao et al., 2017). The possibility of reacting these carbohydrates at temperatures above 150°C in an extruder is highly probable leading to the faster production of oxidation products such as alcohols, acids or other volatile products (Formela et al., 2018).

### Reactive Extrusion of Lignocellulosic Biomass

During compounding of biomass in PLA, hydrolysis of the polymer chains is known to occur (Le Guen et al., 2017; Thumm et al., 2018). A previous study comparing the mechanical behavior of PLA reinforced with protein-based filler and wood flour reported that under accelerated weathering, the protein-based filler hydrolysed the PLA matrix at least twice as fast as a wood flour filler due to the amino-acids produced from protein hydrolysis (Le Guen et al., 2017). Rice husks contain more proteins than wood (Hamdan et al., 1997; Vadiveloo et al., 2009), however, the GPC analysis indicated comparable molecular weights for the PLA compounded with either wood or rice husk powders. This finding ruled out a potential difference solely based on acid hydrolysis of the PLA chains.

In the case of the rice husk compound, the darkening color could be attributed to two chemical reactions:

- Maillard reaction between the amino acids and the carbohydrates (e.g., xylan), that has been previously reported to occur during processing of the rice husks at 180°C (Vegas et al., 2008),
- Carbohydrate oxidation (i.e. caramelization) that is known to form volatile organic molecules (Jiang et al., 2008).

In both cases, the reactions release volatile compounds and water that can explain the instability of the rice husk compounding process and the plasticization of the PLA matrix. Whilst the oxidation reactions also occur with wood powder, the higher reactivity of the rice husk powder compared to the wood powder was attributed to the ball-milling pre-treatment.

## Conclusions

Wood and rice husk powders were blended by twin-screw extrusion with PLA to be used for 3D printing applications. It was found that the mechanical properties of the 3D printed samples at room temperature were mainly driven by the properties of PLA and anisotropy was only influenced by the 3D printing directionality. In addition, the mechanical properties were lowered by the integration of either filler which was attributed to a lowering of inter-strand cohesion in the 3D printed samples. Past the glass transition temperature of the PLA, the fillers enabled the retention of stiffness measured by the storage modulus.

In terms of visual assessment and process stability however, differences were observed and attributed to chemical reactions triggered by the (a) silica content of rice husk and/or (b) chemical reactions including Maillard reaction and carbohydrate oxidations. These chemical reactions were potentially enhanced by a particle size difference (i.e., rice husk powder was smaller and denser than the wood powder) and triggered during the temperature and shearing conditions of the extrusion process. Little polymer research has been conducted on the potential of using reactive biomass filler particles based on mechano-chemistry (i.e., ball milling). These unique findings indicate that biomass can trigger chemical reactions that change the polymer rheological behaviors during processing dramatically and reinforces the need for careful attention when matching a biopolymer matrix with a biomass filler.

The ball-milled rice husk powder's effect on thinning PLA, if controlled during the compounding process, is of major interest in the development of reactive sustainable plastic additives.

## Data Availability Statement

All datasets generated for this study are included in the manuscript/supplementary files.

## Author Contributions

M-JL and CM-L contributed to the design of the research. All authors contributed to its implementation and the analysis of the results as per field of expertise and wrote the manuscript.

### Conflict of Interest

The authors declare that the research was conducted in the absence of any commercial or financial relationships that could be construed as a potential conflict of interest.
